# Mr. Seyer's Case of Fracture

**Published:** 1801-12

**Authors:** J. Syer


### *To the Editors of the Medical and Phyfical Journal.*

GENTLEMEN,

If you fhould think the enclofed Cafes at all worthy the attention of your numerous Readers, they are with due fubmiffion offered to your confideration. The firft is a cafe of violent Concuffion of the Brain, fucceeded by active inflammation of its membranes, which terminated fuccefsfully. The laft is defective, inafmuch as I was unacquainted with the ftate of the patient previous to his death; but is merely the hiftory of a diffection of the dead body: it was a fubject who deftroyed himfelf by drinking ardent fpirits to excefs. It did not fall to my lot to attend the firft cafe till the laft or convalefcent ftage, fo that I can give no minute detail of it, but muft confine myfelf to the general circumftances of it, as they were communicated to me by the refident Surgeon of the Infirmary where the patient was attended.

July 25, 1798. A private foldier, about twenty-four years of age, of a full habit, but previoufly healthy, fell from the top of a loaded baggage waggon upon a ftone pavement, fomewhat in a ftate of intoxication. Being called to the man immediately afterwards, I found the injury was received in the fituation of the lower and pofterior angle of the left parietal bone; it was accompanied with a profufe hæmorrhage from the correfponding ear, and the man was foon conveyed, fenfelefs, to the county hofpital in Kent. Prior to his removal, I had examined the cranium, the wound in the integuments barely admitting the introduction of a common probe. I could not difcover either fracture or depreffion; but as foon as he was put to to bed in the hofpital, the attending Surgeon thought himfelf juftified in making a free incifion in an oblique direction clofe to the wounded part; however, no injury was perceived on the cranium exteriorly. Notwithftanding the comatous ftate of the patient at this ftage of the injury, the leaft preffure upon the fwelling of the furrounding parts induced him to fcream violently, and to utter execrable oaths. As foon as the Surgeon's fingers were withdrawn, the man funk into a profound fleep, attended with ftertor. When he awoke, he was liberally let blood from the arm, and confined to a ftrict afthenic plan; his medicines were of the faline clafs, without any opium; and his diet principally farinaceous. On the following evening he was attacked with violent delirium, and fymptoms indicating acute inflammation of the membranes of the brain. He had now a blifter applied to his head, and was allowed to bleed freely from an incifion made in the temporal artery. In this alarming fituation the patient remained with little variation, under ftrong confinement for twelve or fourteen days, fecured to the bed by ftraps, and watched day and night. He was conftantly raving, and talking to himfelf or his attendants; his eyes prefented a wild ftare, and feemed to project from their orbits; he would appear to dofe at intervals, but his reft during this period was never perfect, fo that the leaft noife would aroufe him. It is a little fingular to relate, that the eye on that fide of the head that fuftained the injury was never clofed during this violent derangement. The ftate of his pulfe, and excretions within this period, or whether the patient had the natural command over the evacuations from the rectum and urinary bladder, are points that I am utterly at a lofs to communicate. With regard to the bafis of his medical treatment, he was found to derive the principal benefit from frequent bleeding from the temporal artery, and the occafional repetition of blifters to the head. The delirium fubfided gradually, but the irritability which followed it, was denoted by an impatience of contradiction, ftate of univerfal tremor; and frequently during recovery, he experienced flight paroxyfms of mental derangement until Auguft 19, when he completely regained his intellects. The laft time the operation of bleeding from the temporal artery was performed, he experienced an obftinate hæmorrhage afterwards, in confequence of ftruggling, and friction againft the bed-cloaths; probably this artery was not entirely divided, as it is obferved in general to retract readily enough after this operation, where the incifion is freely made.

After a month's confinement in the hofpital he returned to his regiment, (then quartered about eighteen miles off) in a very debilitated ftate. For many days he was affected with univerfal universal tremor whilst seated, and had obviously lost much of the energy of his mind. His countenance was pale, but not much emaciated; his pulse, for a considerable time, was feeble, but otherwise tolerably regular; and his appetite for food was moderately good. He was in the habit of taking, under my care, a strong decoction of cinchona with the acid elixir of vitriol, until his final recovery, which was established in about three weeks from his release from the Infirmary. Having attended to this case at first with much apprehension about its final event, I felt a very lively interest in the man's welfare, and thought myself fortunate in procuring, through the kindness of the house surgeon to the Infirmary, even the incomplete history of his case, such as my notes have enabled me to produce.

Before I conclude, I would beg leave to add, that the original situation of this patient corresponded with what an attentive practitioner will have frequent occasion to observe in injuries of the head, which are mostly complicated; viz. that the common symptoms of concussion were so strong at the first period of his indisposition, that the pulse was slow and oppressed, and that great coldness pervaded the upper and lower extremities. I have made no mention of any purgative plan being pursued, which no doubt was discretionally interposed; and which the celebrated Dr. Fordyce lays great stress upon in cases of inflammation of the brain from external violence. In bringing forward this case, I am aware that it will not elicit any new doctrine, but merely confirm the evidence of others that may be associated with it, of the striking good effects of blisters, and arteriotomy from a part contiguous to the injury.

### Case II.

I could gather but few particulars respecting the situation of the unfortunate subject of this case prior to his death. He was a robust young Irish Dragoon, and I was accidentally requested by the inspecting physician of the district who had attended him during his confinement, to assist in examining the dead body. The poor man had complied with the example of several of his fellow soldiers in drinking large quantities of strong spirits, who all, except himself, had escaped with impunity. Soon after finishing the last potion, I understood that he became very comatous, in which state he remained, incapable of speech, till the fourth day, when he expired. He had been let blood once or twice very freely, and had been forced to take some strong cathartics, but swallowed very imperfectly; in fact, nothing alleviated him in the smallest degree. The dead body, which was inspected the following day, had a dark purple hue, with marks of of partial extravasation about the head. Having made a horizontal section of the ossa cranii, it was observed that the dura mater was very much crouded with vessels carrying red blood, and upon its upper surface, in the direction of its proper blood vessels, were seen a number of small excrescences of a glandular appearance. This membrane no where adhered to the subjacent tunics, but the pia mater was extremely vascular, and upon the entire surface of the tunica arachnöides was found a thick stratum of true pus. The tunica arachnöides and pia mater were in close contact, so that we could distinguish no natural separation, but they adhered as one membrane to the cerebrum. The ventricles were exposed, but no preternatural appearance of pus or redundant serum occurred. The plexus choroides was paler and less vascular to the eye than usual; we also remarked, that the glandula pinealis was larger than ordinary, and contained proportionably more earthy matter. The membranes of the cerebellum were examined, but nothing remarkable was observed, its investing membranes shewed no mark of secreted pus; and the origin of the medulla spinalis looked healthy. The pupils of the eyes were extremely dilated, and his sensibility was nearly extinguished soon after taking the spirits. It may be worth while to observe, that during his confinement he was frequently seen to rub his forehead and the epigastric region, as if to point out in some measure the sources of his suffering. We next examined the abdominal viscera. The liver appeared free from disease, and the gall bladder was nearly full of bile. The patient having taken scarcely any nutriment during his confinement, the stomach was found quite empty; but upon making an opening into it through its whole extent, its villous internal coat was highly vascular, exhibiting strong vestiges of recent inflammation. The beginning of the duodenum assumed nearly the same appearances. The small intestines were much collapsed, and the peritoneal covering was observed to be highly injected, as it were, by the inflammation; as was likewise a portion of the colon on the right side. We lastly examined the thoracic viscera: All that we remarked here, was an unusual florid appearance of the pleura covering the aorta and the pulmonary artery. Upon opening the heart, we found its auricles and ventricles loaded with a substance of an intermediate nature between the coagulating lymph of the blood and a medullary fat. The lungs were free from disease, and did not appear to have sympathized with the other parts of the system.

Having recited the particulars of this dissection as they were noted immediately afterwards, in the summer of the year 1798, I cannot help suggesting that few cases are recorded in common, mon, which afford, from the fame caufe, fo ftriking an inftance of active inflammation. One effential point this cafe appears to controvert, which now infinuates itfelf into the modern doctrine of medical men, namely, the fedative effects, or preconceived indirect debility, generally attributed to the diffufible ftimulus of fpirituous liquors. It is difficult, in compliance with this theory, to explain the morbid effects of this active poifon. Had this patient, in the firft inftance, taken a ftrong emetic, and warm watery diluents been largely adminiftered, there is a probability that the mifchief might have been in great meafure arrefted: Still, however, cafes that have terminated like the prefent, fatally, are not wanting to demonftrate the inefficacy of our art, even when affifted by the moft enlightened human reafon.

I am, &c.

J. SYER.

